# A type 2 diabetes patient with three years of persistent abdominal pain: the culprit was variegate porphyria—a case report

**DOI:** 10.3389/fendo.2026.1777122

**Published:** 2026-02-27

**Authors:** XiaoLi Yan, ZhongSen Xu, ZiYing Huang, JinSen Chen, MengTing Ye, YuLin Huang, ChenChen Wang

**Affiliations:** 1 Department of Endocrinology, Quzhou KeCheng People’s Hospital, Quzhou, Zhejiang, China; 2 Department of Pharmacy, Quzhou KeCheng People’s Hospital, Quzhou, Zhejiang, China

**Keywords:** case report, diabetic neuropathy, neurovisceral symptoms, *PPOX* gene, variegate porphyria

## Abstract

**Introduction:**

Variegate porphyria (VP) is a rare metabolic disorder. Its diagnosis is challenging when cutaneous features are absent and symptoms overlap with common conditions like diabetic neuropathy.

**Case presentation:**

We report a 71-year-old female with a 30-year history of type 2 diabetes and a 3-year history of mild chronic abdominal pain and psychiatric symptoms. Extensive workup for common abdominal pathologies was negative. A positive urine sun exposure test prompted genetic analysis, which identified a heterozygous pathogenic variant in the *PPOX* gene (c.567A>C, p.Gln189His), confirming VP. Her mild acute attack was managed successfully with intravenous glucose and safe psychotropic agents, alongside adjusted glycemic targets to prevent catabolism.

**Conclusion:**

This case underscores that VP can present atypically without skin lesions. It highlights the importance of considering VP in diabetic patients with unexplained neurovisceral symptoms and demonstrates that mild attacks can be managed with tailored supportive care.

## Introduction

Porphyria is a group of rare metabolic disorders caused by abnormal enzyme activity during heme biosynthesis. The heme biosynthesis pathway comprises eight enzymatic steps. PPOX (protoporphyrinogen oxidase) catalyzes the seventh step. Heterozygous loss-of-function mutations in the *PPOX* gene result in significantly reduced enzyme activity. This leads to the accumulation of its substrate, protoporphyrinogen IX, and the upstream porphyrin precursors, coproporphyrinogen III. Additionally, due to impaired negative feedback regulation, the earlier water-soluble precursors, δ-aminolevulinic acid (ALA) and porphobilinogen (PBG), may also increase (1). The accumulation of these metabolites is responsible for the clinical manifestations of the disorder. Patients typically experience acute neurovisceral attacks, characterized by severe abdominal pain, autonomic dysfunction, and neurological or psychiatric symptoms. In a subset of patients, chronic cutaneous photosensitivity and an increased risk of hepatobiliary complications are also observed. This specific clinical and biochemical phenotype, resulting from PPOX deficiency, is classified as variegate porphyria (VP) (2). We herein report a rare case of VP in an elderly patient with comorbid diabetes mellitus. Due to the absence of the typical cutaneous manifestations, the patient’s abdominal pain and psychiatric symptoms were misattributed to diabetic neuropathy for approximately three years. The definitive diagnosis was ultimately established through genetic testing. This case underscores the importance of considering VP in the differential diagnosis of unexplained neurovisceral symptoms, even in the absence of skin lesions, particularly among patients with diabetes.

## Case description

A 71-year-old woman presented three years ago with diffuse abdominal discomfort and bloating without apparent cause, accompanied by insomnia. After consultation at a local hospital, she was diagnosed with anxiety disorder and treated with zopiclone and escitalopram. However, the abdominal discomfort persisted and recurred, leading to ten subsequent hospitalizations at our institution. The patient has a 30-year history of diabetes mellitus. Initially treated with oral hypoglycemic agents, insulin therapy was initiated 14 years ago due to inadequate glycemic control. 8 years ago, the patient developed numbness in the extremities and occasional foot spasms, leading to a diagnosis of diabetic peripheral neuropathy.

**Figure 1:**
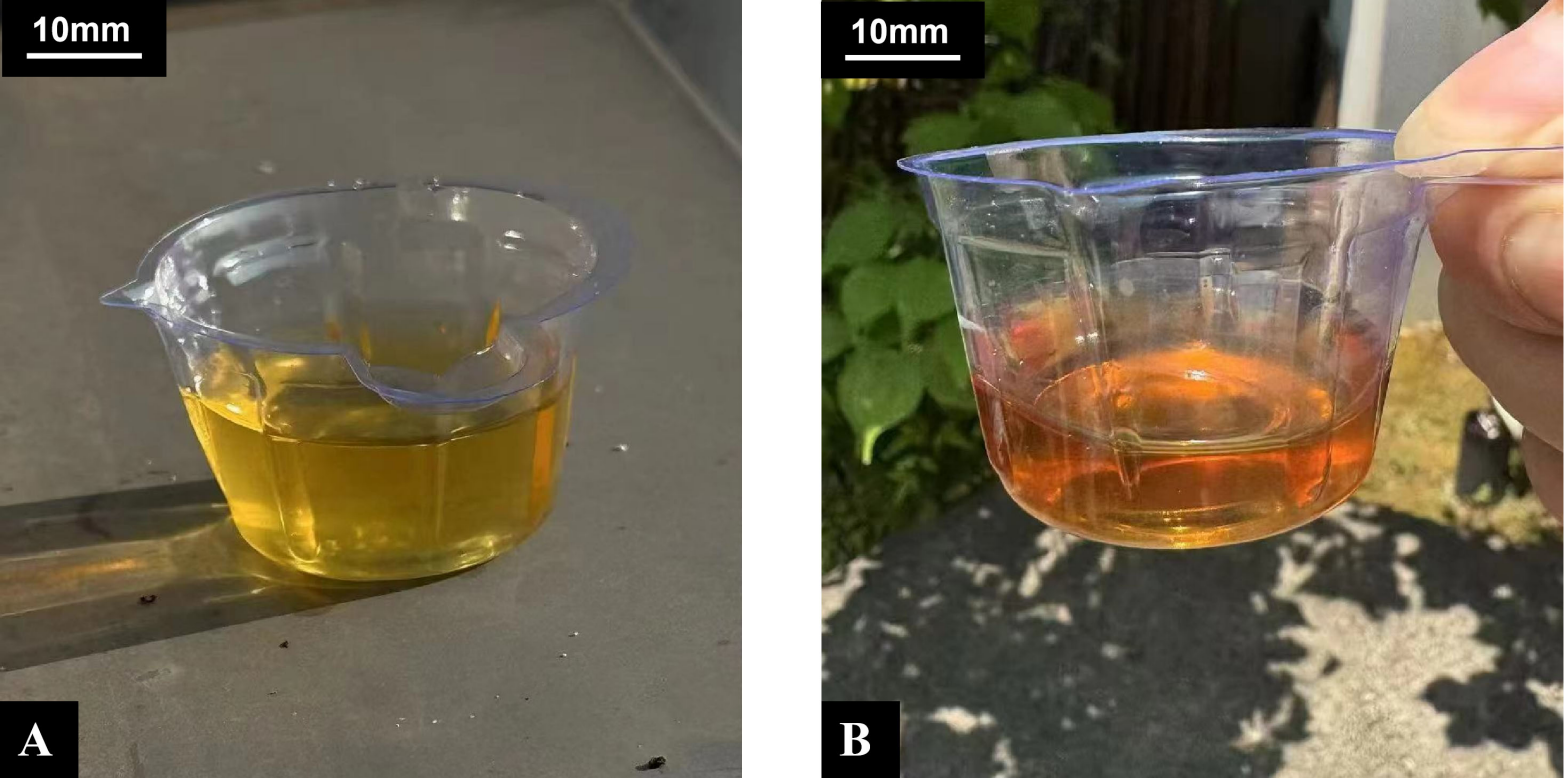
Non-standardized sunlight exposure test for urinary porphyrin precursors. The image shows the change in urine color from yellow **(A)** to wine red **(B)** after approximately 30 minutes of exposure to direct ambient sunlight. A scale marker (10 mm) is included for size reference. This qualitative test lacks sensitivity and specificity and served only as an initial screening cue in a resource-limited setting; it is not a diagnostic tool for variegate porphyria. Definitive diagnosis requires genetic or standardized biochemical confirmation (see Discussion and Limitations).

**Figure 2:**
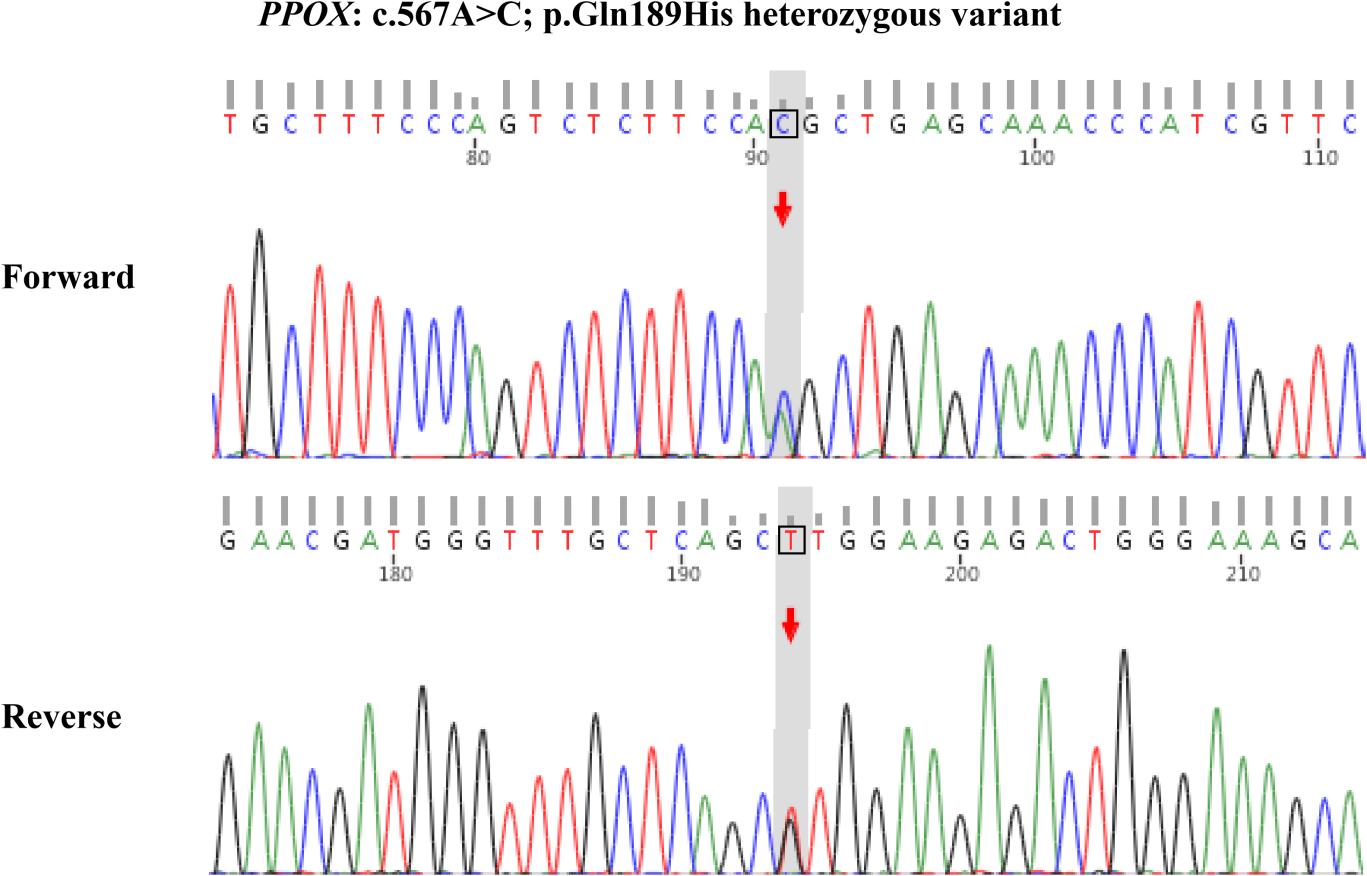
Gene sequence of the patient.

The patient’s primary complaint during this hospitalization remains persistent abdominal distension and pain throughout the abdomen (NRS = 2), accompanied by muscle weakness. Concurrently, psychiatric symptoms have worsened compared to previous episodes, manifesting as: depressed mood, diminished interest, accompanied by anxiety, worry, rapid heartbeat, chest tightness, occasional feelings of impending doom, and poor sleep. The patient’s fasting blood glucose level was 6.52 mmol/L (reference range 3.9-6.1 mmol/L, glucose oxidase method). Urine ketones were negative, ruling out ketoacidosis, other serological indicators as shown in **Table 1**. The patient’s abdominal ultrasound and CT scan revealed no gallbladder stones, cholecystitis, common bile duct stones, gastrointestinal perforation, acute pancreatitis, intra-abdominal abscess, mesenteric artery occlusion, or intestinal obstruction. Additionally, endoscopic examination of the stomach, duodenum, and colon revealed no ulcers, tumors, ischemia, bleeding, or inflammation. Given the patient’s abdominal discomfort and fatigue, combined with psychiatric symptoms, porphyria was considered. Since quantitative urinary PBG testing was unavailable at our hospital, a non−standardized sunlight exposure test was performed as an initial screening measure, resulting in a color change from yellow to wine red (**Figure 1**). This test is neither sensitive nor specific for VP and cannot differentiate between porphyria types; it served only to raise clinical suspicion, prompting referral for definitive genetic analysis. Genetic analysis of the patient revealed a heterozygous variant in the *PPOX* gene. The variant was identified at chromosomal position chr1:161138317 (GRCh37/hg19), corresponding to exon 6 of 13 in transcript NM_001122764.3. The specific nucleotide change is c.567A>C, resulting in a missense substitution at the protein level: p.Gln189His (glutamine to histidine at codon 189). Bidirectional Sanger sequencing (forward and reverse) confirmed the heterozygous state of this variant in the patient, as illustrated in the accompanying electrophoretograms (**Figure 2**). The results were consistent between both sequencing directions, validating the presence of the c.567A>C alteration in a heterozygous manner. This variant is classified as pathogenic in ClinVar and is extremely rare in population databases (gnomAD frequency 0.00000398). In−silico predictors (PolyPhen−2, SIFT, CADD) unanimously support a deleterious effect, and it has been previously reported in patients with variegate porphyria (3, 4). Therefore, the patient was diagnosed with Variegate Porphyria. To further assess the severity of the acute episode, key clinical and laboratory parameters were evaluated. Serum sodium was 144 mmol/L (within normal range), ruling out hyponatremia. Although the patient had chronic psychiatric symptoms and subjective muscle weakness, her abdominal pain was mild (NRS = 2) and not associated with nausea, vomiting, ileus, or progressive neurological deficits. Based on current management guidelines for acute porphyrias (5, 6), the absence of severe pain, autonomic instability, hyponatremia, or neurological ‘red flags’ supported the classification of this episode as a mild acute attack.

**Table 1:** Laboratory investigations.

Test	Result	Reference range
WBC	6.3×10^9^/L	3.5-9.5×10^9^/L
EO	0.02×10^9^/L	0.02-0.52×10^9^/L
CRP	10.1 mg/L	0-10 mg/L
TBIL	12.2 μmol/L	≤ 23.0 μmol/L
AMY	32 U/L	35-135 U/L
Cr	55 μmol/L	41-81 μmol/L
D-dimer	0.91 mg/L	0-0.60 mg/L
Blood lead	26.9 μgl/L	< 200 μgl/L
Sodium	144 mmol/L	135-145 mmol/L

WBC, White Blood Cell; EO, Eosinophil; CRP, C-reactive protein; TBIL, Total Bilirubin; AMY, Amylase; Cr, Creatinine.

**Figure 3:**
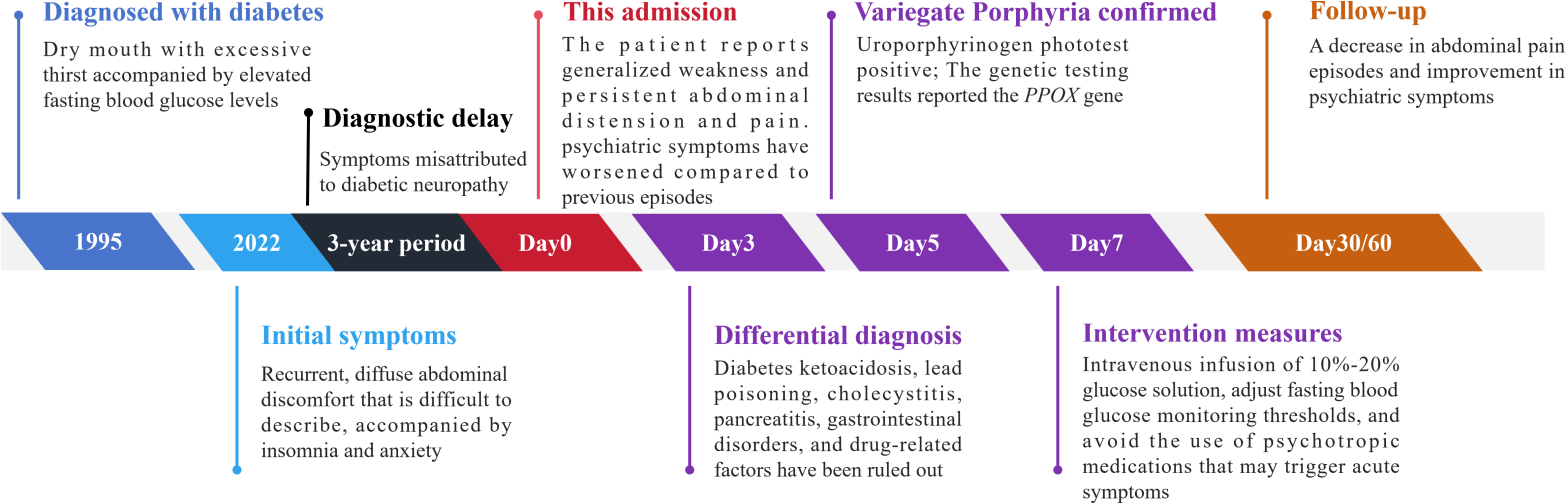
Clinical timeline highlighting diagnostic delay and management.

Due to the unavailability of hemoglobin, the treatment plan was adjusted to intravenous infusion of 10%-20% glucose solution (dissolved in saline) with insulin added proportionally. Following the initiation of intravenous 10–20% glucose infusion, the patient’s abdominal pain decreased within 24–48 hours, and her neuropsychiatric symptoms (anxiety, low mood, insomnia) gradually improved over the subsequent days. This rapid response to carbohydrate loading is consistent with the pathophysiology of an acute porphyric attack, wherein glucose suppresses hepatic ALAS1 activity and reduces the accumulation of neurotoxic precursors. Given the patient’s concomitant diabetes, fasting blood glucose was elevated and maintained at 8-9 mmol/L during hospitalization to avoid acute symptoms triggered by calorie and carbohydrate restrictions. Treatment plans and medication selection for patients’ psychiatric symptoms refer to the Porphyria Foundation website (https://porphyriafoundation.org/) and the European Porphyria website (https://www.porphyria-europe.org/). Specifically, lorazepam 1mg once daily is recommended to improve sleep and anxiety, and paroxetine 10mg once daily is recommended for antidepressant effects. During follow-up, thorough disease education was provided to the patient regarding the importance of adequate caloric intake for disease management and adjustments to fasting blood glucose monitoring thresholds. The patient reported a decrease in abdominal pain episodes and improvement in psychiatric symptoms at both the three-month and six-month follow-ups (≤ once per month). The patient's medical history and the diagnostic and treatment process after admission are shown in **Figure 3**.

## Discussion

The clinical presentation of VP is highly variable and nonspecific, leading to frequent misdiagnosis (7). Our patient, with a 30-year history of diabetes mellitus, presented with a 3-year history of persistent but mild abdominal discomfort (without nausea or vomiting), accompanied by insomnia, anxiety, and depression. The absence of bullous skin lesions typically associated with photosensitivity initially diverted the diagnostic suspicion away from a porphyria. Consequently, her symptoms were primarily attributed to diabetic peripheral neuropathy. However, due to their chronic nature and association with neuropsychiatric features, urine sunlight exposure test was performed during hospitalization, which returned positive. It is important to note that the urine sunlight exposure test used in this case is not a validated diagnostic tool. It lacks standardization, has variable sensitivity and specificity, and cannot distinguish VP from other acute porphyrias. Its utility is limited to settings where specialized biochemical testing is inaccessible, and it should always be followed by confirmatory genetic or quantitative porphyrin profiling (5, 8). The diagnosis of VP was subsequently confirmed by genetic analysis identifying a pathogenic *PPOX* variant. The identified variant, *PPOX* c.567A>C (p.Gln189His), is a known pathogenic mutation and has been previously reported in patients with VP (3, 4). Applying ACMG guidelines (9), it is classified as Pathogenic (criteria: PS1, PM1, PM2, PP3, PP4). It is absent in population cohorts (gnomAD frequency <0.00001) and is predicted to disrupt enzyme function by multiple in−silico tools. Prior reports have linked this mutation to VP phenotypes, confirming its role in heme pathway dysregulation (3, 4). In our patient, the heterozygous state of this variant correlates with the partial enzyme deficiency characteristic of VP, reinforcing the genetic diagnosis despite the lack of quantitative porphyrin profiling at our center.

To systematically exclude other common etiologies of chronic abdominal pain, a comprehensive serological evaluation was performed. The patient’s normal white blood cell (WBC) and only marginally elevated C-reactive protein (CRP) levels argued against an active infectious or significant inflammatory process (e.g., intra-abdominal abscess, severe cholecystitis, or inflammatory bowel disease). The absence of eosinophilia did not support a parasitic infection or eosinophilic gastroenteritis. Normal serum amylase (AMY) and total bilirubin (TBIL) levels effectively ruled out acute pancreatitis and acute biliary obstruction, respectively. Renal function, as indicated by creatinine (Cr), was within normal limits, excluding uremic causes of gastrointestinal symptoms. While the D-dimer level was slightly elevated, this finding, in the absence of clinical signs or imaging evidence of mesenteric ischemia or venous thrombosis, was considered nonspecific and possibly related to chronic low-grade inflammation or immobility. Importantly, the blood lead level was within the normal range, effectively excluding lead poisoning—a known mimicker of acute porphyria due to its inhibition of ALA dehydratase (10). This pattern of largely unremarkable routine serology, in the context of extensive negative abdominal imaging and endoscopy, effectively narrowed the differential diagnosis. The convergence of negative findings for common abdominal pathologies heightened the clinical suspicion for a metabolic disorder such as porphyria, which was subsequently confirmed by genetic testing.

VP is a rare autosomal dominant disorder caused by mutations in the *PPOX* gene, which is crucial in the heme biosynthesis pathway. This disorder is characterized by a partial deficiency of protoporphyrinogen oxidase, leading to the accumulation of porphyrins and their precursors, which manifest as cutaneous photosensitivity and acute neurovisceral attacks (3, 4). The genetic heterogeneity of VP is well-documented, with numerous mutations identified in the *PPOX* gene, including small deletions, splicing defects, nonsense, and missense mutations, which contribute to the variability in clinical presentation (4, 11). The clinical manifestations of VP are diverse, ranging from skin lesions in sun-exposed areas to severe neuropsychiatric symptoms during acute attacks. This patient presented with an atypical and relatively subtle VP phenotype, characterized by only mild abdominal pain (NRS ≤ 2), psychiatric symptoms (insomnia, anxiety, depression), and no bullous skin lesions. Urinary discoloration occurred only upon sun exposure, further contributing to the diagnostic obscurity. Critically, the patient had a 30-year history of diabetes mellitus and an 8-year history of diabetic peripheral neuropathy. It is well-established that long-standing diabetes can lead to severe peripheral, autonomic, and even central neuropathy, often manifesting with gastrointestinal symptoms such as abdominal pain, bloating, and constipation (12–14). The significant overlap between these diabetic complications and the neurovisceral manifestations of VP likely created a diagnostic blind spot, where the patient’s symptoms were readily attributed to her pre-existing diabetic neuropathy.

Based on the established understanding of acute porphyrias, a reduction in carbohydrate intake, through fasting, dieting, or illness is a well-known precipitating factor for acute neurovisceral attacks (8). The pathophysiological mechanism involves the glucose-mediated downregulation of the rate-limiting hepatic enzyme 5-aminolevulinic acid synthase 1 (ALAS1), a process mediated via peroxisome proliferator-activated receptor gamma coactivator 1-alpha (PGC-1α). In a carrier of a heterozygous *PPOX* mutation, decreased carbohydrate intake can thus lead to the derepression of ALAS1, increased flux through the heme biosynthesis pathway, and consequent accumulation of neurotoxic precursors (15). In the present case, the patient’s long-standing type 2 diabetes likely involved chronic, medically advised modifications to carbohydrate intake, which may have constituted a persistent metabolic stressor and contributed to the provocation of symptoms. This specific trigger remained unrecognized, as her non-classical presentation (lacking cutaneous lesions) and overlapping symptomatology with diabetic complications led to a three-year diagnostic delay.

The contemporary management of acute hepatic porphyria (AHP), including VP, is stratified according to attack severity and follows established guidelines. For moderate to severe acute neurovisceral attacks which characterized by intense pain, nausea/vomiting, hyponatremia, or neurological signs - prompt administration of intravenous hemin (e.g., haem arginate) is the cornerstone of treatment, acting to repress hepatic ALAS1 activity and reduce the production of neurotoxic precursors (1, 16). Supportive care, including aggressive analgesia with safe opioids, management of autonomic symptoms, and strict avoidance of porphyrinogenic agents, is imperative (5). For mild attacks, initial management focuses on eliminating precipitating factors and providing sufficient caloric intake. The patient’s symptom improvement following intravenous glucose supports a porphyric etiology rather than diabetic neuropathy alone. In diabetic autonomic neuropathy, symptom response to carbohydrate loading is not expected, whereas in acute porphyrias, glucose administration is a well-established mechanism to alleviate neurovisceral symptoms by downregulating ALAS1. This temporal and mechanistic correlation reinforces the diagnosis of VP as the primary driver of her acute presentation (5, 6). The treatment decision in this case was based on a comprehensive clinical assessment of attack severity. The patient presented with mild abdominal pain (NRS ≤ 2), no gastrointestinal complications, normal serum sodium (144 mmol/L), and an absence of neurological or autonomic ‘red flag’ signs. Guidelines recommend that such mild acute attacks be initially managed by eliminating precipitating factors and providing adequate carbohydrate/glucose loading to suppress ALAS1 activity (5, 6, 15). Therefore, after detailed discussion of the risks, benefits, and alternatives—including hemin therapy (typically indicated for moderate−to−severe attacks)—a joint decision was made with the patient to forgo hemin and initiate intravenous glucose supplementation. This approach led to the timely resolution of her abdominal symptoms and gradual improvement in her neuropsychiatric condition, confirming its suitability for this mild attack. This approach aligns with the principle of tailoring intervention to attack severity. For her comorbid psychiatric symptoms (anxiety and depression), medication selection was meticulously guided by resources from the Porphyria Foundation and the European Porphyria Network to ensure the use of agents considered safe in AHP, thereby avoiding inadvertent provocation of further attacks.

Regarding long-term management and prevention of recurrence, maintaining adequate caloric intake is paramount, as fasting or carbohydrate restriction is a well-known trigger. Given the patient’s co-existing diabetes mellitus, a careful balance was required. To avoid a catabolic state that could precipitate porphyric symptoms, her inpatient fasting blood glucose target was temporarily adjusted to a permissible range of 8–9 mmol/L, deviating from stricter diabetic control during the acute phase. She received comprehensive education on the importance of regular meals and avoiding prolonged fasting. All aspects of this personalized management plan were thoroughly discussed and agreed upon by the patient. The efficacy of this strategy is supported by her follow-up reports at three and six months, during which she noted a marked reduction in the frequency of symptomatic episodes (to ≤1 per month) and a significant improvement in her overall quality of life.

## Conclusion

This case illustrates the diagnostic challenge of VP in an elderly patient with long-standing type 2 diabetes mellitus. The absence of cutaneous photosensitivity and the overlapping symptomatology with diabetic neuropathy led to a three-year diagnostic delay. Definitive diagnosis was achieved through genetic testing, revealing a heterozygous *PPOX* c.567A>C (p.Gln189His) variant. The patient’s mild acute attack was successfully managed with intravenous glucose and safe psychotropic agents, highlighting the importance of tailored therapy based on attack severity. Long-term management focused on preventing catabolic states, which necessitated a temporary relaxation of glycemic targets during the acute phase. This integrated approach resulted in a significant reduction of symptoms and improved quality of life during follow-up.

### Key clinical takeaways

#### For endocrinologists

VP should be considered in the differential diagnosis of patients with diabetes who present with chronic, unexplained abdominal pain and neuropsychiatric symptoms, even in the absence of skin lesions.

#### Diagnostic strategy

In resource−limited settings where quantitative PBG/porphyrin testing is unavailable, a non−specific screening test such as urine sunlight exposure may raise clinical suspicion, but it must be followed by confirmatory genetic testing or standardized biochemical assays for accurate diagnosis and classification of porphyria.

#### Management of comorbidity

Managing VP in patients with diabetes requires a careful balance. Avoiding fasting and maintaining adequate caloric intake is paramount to prevent attacks, which may justify transiently modified glycemic targets (e.g., fasting glucose 8–9 mmol/L) during symptomatic periods to avoid catabolism.

#### Therapeutic triage

Not all acute porphyria attacks require hemin infusion. Mild attacks without severe symptoms, autonomic instability, or hyponatremia can be effectively managed with carbohydrate loading and supportive care after shared decision-making with the patient.

### Limitations

This report describes a single case from a single center. The positive response to a urine sunlight exposure test, while suggestive, is not a standardized quantitative diagnostic method for VP and lacks specificity. Definitive diagnosis should rely on genetic testing and/or validated porphyrin profiling. However, quantitative porphyrin profiling (e.g., urinary PBG and porphyrin HPLC) was not performed due to local resource constraints. Future evaluations in similar settings may benefit from collaboration with referral centers for confirmatory biochemical testing. Formal autonomic function testing was not performed, which could have provided objective evidence of autonomic involvement and helped further differentiate between diabetic and porphyric neuropathy. Future studies in similar patients may benefit from incorporating standardized autonomic assessments. The patient’s sustained clinical improvement, though encouraging, was assessed over a relatively short follow-up period of six months. Longer-term monitoring is required to fully evaluate the stability of this management strategy and to screen for potential late complications of VP, such as hepatocellular carcinoma.

## Data Availability

The original contributions presented in the study are included in the article/supplementary material. Further inquiries can be directed to the corresponding author.
